# A phenomenological model of the X-ray pulse statistics of a high-repetition-rate X-ray free-electron laser

**DOI:** 10.1107/S2052252523008242

**Published:** 2023-10-03

**Authors:** Trey W. Guest, Richard Bean, Raimund Kammering, Grant van Riessen, Adrian P. Mancuso, Brian Abbey

**Affiliations:** a La Trobe Institute for Molecular Science, La Trobe University, Bundoora, VIC 3086, Australia; b Department of Mathematical and Physical Sciences, School of Engineering, Computing and Mathematical Sciences, La Trobe University, Bundoora, VIC 3086, Australia; c European XFEL, Holzkoppel 4, 22869 Schenefeld, Germany; d Deutsches Elektronen-Synchrotron, Notkestraße 85, 22607 Hamburg, Germany; e Diamond Light Source, Harwell Science and Innovation Campus, Didcot, OX11 0DE, UK; Brookhaven National Laboratory, USA

**Keywords:** X-ray free-electron lasers, XFELs, correlated fluctuations, time-resolved studies, computational modelling, dynamic simulations, MHz XFELs, wavefront propagation, temporal coherence

## Abstract

Many coherent imaging applications that utilize ultrafast X-ray free-electron laser (XFEL) radiation pulses are highly sensitive to fluctuations in the shot-to-shot statistical properties of the source. Understanding and modelling these fluctuations are key to successful experiment planning and necessary to maximize the potential of XFEL facilities. Current models of XFEL radiation and their shot-to-shot statistics are based on theoretical descriptions of the source and are limited in their ability to capture the shot-to-shot intensity fluctuations observed experimentally. The lack of accurate temporal statistics in simulations that utilize these models is a significant barrier to optimizing and interpreting data from XFEL coherent diffraction experiments. Presented here is a phenomenological model of XFEL radiation that is capable of capturing the shot-to-shot statistics observed experimentally using a simple time-dependent approximation of the pulse wavefront. The model is applied to reproduce non-stationary shot-to-shot intensity fluctuations observed at the European XFEL, whilst accurately representing the single-shot properties predicted by FEL theory. Compared with previous models, this approach provides a simple, robust and computationally inexpensive method of generating statistical representations of XFEL radiation.

## Abstract 2

A simple, robust and computationally inexpensive method of generating statistical representations of X-ray free-electron laser (XFEL) radiation is presented.

## 1. Introduction

X-ray free-electron lasers (XFELs) produce femtosecond duration X-ray wavelength pulses with high spatial coherence and spectral brightness (Buts *et al.*, 2006 ▸). These properties are ideally suited for coherent X-ray diffraction applications and have enabled the observation of nanoscale features and processes relevant to a broad range of scientific applications (Martin *et al.*, 2020 ▸; Lehmkühler *et al.*, 2021 ▸; Vassholz *et al.*, 2021 ▸). For many of these applications, high spatial and temporal resolution can only be achieved by merging a large number of individual diffraction or scattering measurements (Chapman *et al.*, 2006 ▸; Lehmkühler *et al.*, 2020 ▸; Bielecki *et al.*, 2020 ▸). In serial XFEL experiments, diffraction patterns are integrated over many single-shot exposures of a reproducible sample or process (Chapman *et al.*, 2011 ▸; Loh & Elser, 2009 ▸). This mode of experiment can enable the study of samples that would otherwise produce diffraction patterns too photon-sparse to extract useful information at high resolution and is essential to the study of ultrafast processes and soft matter (Coe & Fromme, 2016 ▸).

The quality of the diffraction patterns obtained when merging multiple exposures of a sample is sensitive to the shot-to-shot statistics of the irradiating wavefields (Nugent, 2011 ▸). Understanding the characteristics of these wavefields and their shot-to-shot temporal statistics is a persistent challenge in successfully performing and optimizing serial XFEL experiments (Vartanyants *et al.*, 2011 ▸; Seaberg *et al.*, 2019 ▸; Lee *et al.*, 2020 ▸). Poor shot-to-shot correlation in the phase and intensity of the irradiating pulses leads to exposure conditions that fluctuate in time and subsequently reduce the contrast of time-integrated diffraction measurements (Williams *et al.*, 2007 ▸; Latychevskaia *et al.*, 2015 ▸). These correlations can evolve on timescales far exceeding the duration of a single pulse and are in many cases not fully captured by current descriptions of the FEL process alone.

The lack of available tools to observe and model fluctuations in XFEL wavefield statistics has been identified as a limiting factor across a broad range of coherent X-ray imaging applications (Nagaya *et al.*, 2016 ▸; Reddy *et al.*, 2017 ▸; Nakano *et al.*, 2018 ▸; Cao *et al.*, 2020 ▸). The interpretation of experimental outcomes in these applications requires an understanding of the sensitivity of key experimental parameters to shot-to-shot fluctuations in the irradiating wavefield (Dallari *et al.*, 2021 ▸). Estimates of these parameters and their sensitivity to fluctuations in pulse characteristics can typically only be evaluated in simulations (E *et al.*, 2021 ▸). The accuracy of these simulations requires that descriptions of the X-ray pulse statistics in the plane of incidence with the sample accurately depict the shot-to-shot pulse statistics that are observed experimentally.

Current methods of determining the statistical properties of XFEL radiation rely on representations of the XFEL source obtained from simulations based on fundamental FEL theory (Reiche, 1999 ▸; Saldin *et al.*, 1999 ▸). These methods provide a route to simulating the characteristics of XFEL radiation by solving the equations of motion for large numbers of electrons (Tran & Wurtele, 1989 ▸) and require a diverse range of physical phenomena to be represented (Agapov & Geloni, 2013 ▸). While these simulations can provide accurate descriptions of the properties of XFEL radiation, such as the self-amplified spontaneous emission (SASE) characteristics of the undulator (Agapov *et al.*, 2014 ▸), they are limited in their capacity to describe fluctuations in pulse characteristics that arise due to non-fundamental effects. These fluctuations can be the result of operational factors and lead to instability of the electron-bunch phase space (Kongtawong *et al.*, 2022 ▸). They are often difficult to characterize (Hellert *et al.*, 2017*a* ▸) and can vary dramatically over the time frame of an experiment (Hellert *et al.*, 2017*b* ▸). In order to predict and interpret the outcome of experiments at FEL facilities, new models of the temporal characteristics of their radiation must be developed that are capable of describing the practical effects of these instabilities on the shot-to-shot properties of the XFEL pulse wavefront.

In this paper, we present a phenomenological model of XFEL radiation that describes shot-to-shot fluctuations of the XFEL wavefront. Unlike current models based on fundamental FEL theory, our description of the shot-to-shot wavefront characteristics captures fluctuations in pulse statistics observed experimentally. In contrast to earlier models, the approach described and demonstrated here does not require a detailed understanding of the operational conditions of the electron beam or undulator. Our phenomenological model instead takes as its inputs the pulse statistics which can be determined from intensity measurements made during an experiment. Using fluctuations in the size and centre of mass of intensity distributions recorded at megahertz (MHz) repetition rates, we apply this model to simulate the shot-to-shot wavefront characteristics of the SPB/SFX instrument at the European XFEL. The results from our model demonstrate the ease and effectiveness of our approach in replicating the temporal characteristics of the XFEL wavefield over a range of key experimental timescales.

## 2. Shot-to-shot intensity statistics at the European XFEL

The European XFEL is the world’s first high-repetition-rate XFEL facility (Decking *et al.*, 2020 ▸). By delivering pulses at sub-microsecond intervals, it has dramatically increased rates of data collection in experiments using XFEL radiation. The advantages of this new mode of operation have already been exploited for coherent imaging applications and have led to the development of time-resolved imaging techniques for the study of dynamically evolving nanoscale systems (Vagovič *et al.*, 2019 ▸; Sobolev *et al.*, 2020 ▸; de Wijn *et al.*, 2022 ▸; Koliyadu *et al.*, 2022 ▸; Holmes *et al.*, 2022 ▸). Pulses at the European XFEL are delivered to beamlines at a megahertz repetition rate in a burst mode of operation (Fröhlich *et al.*, 2019 ▸), where pulse trains containing up to 352 pulses separated by sub-microsecond time delays (minimum 220 ns) are repeated at 10 Hz.

**Figure 1:**
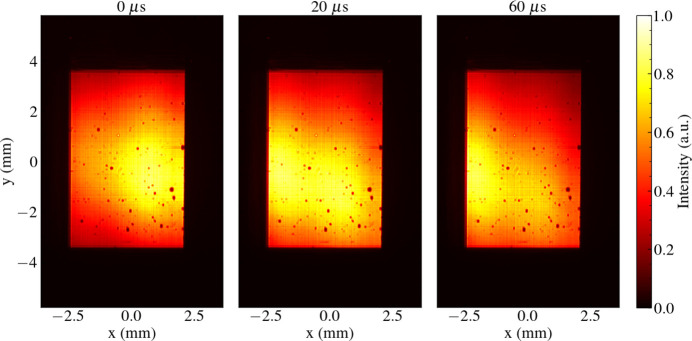
Example spatial intensities of 6.0 keV XFEL pulses on the SPB/SFX instrument at the European XFEL at various delay times starting from the beginning of the pulse train. The intensities shown are averaged over all 100 pulse trains and illustrate a systematic right-to-left drift of the pulse intensity profile on the microsecond timescale. As a result of this drift, the centre of mass of the beam fluctuates within a peak-to-peak range of 700 µm in the horizontal direction and 400 µm in the vertical direction. This drift in the pulse intensity is periodic at 10 Hz. Dark spots distributed across the beam profile are due to surface contamination of the upstream focusing optics.

**Figure 2:**
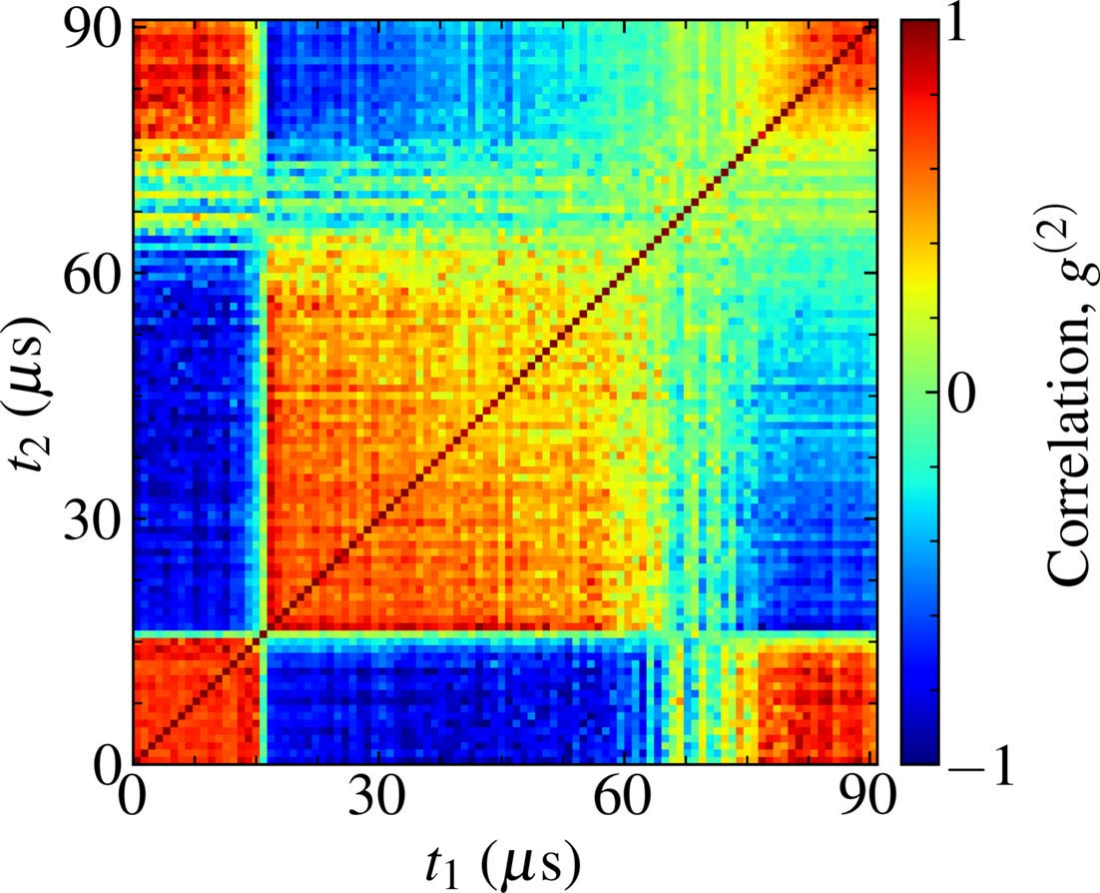
The two-time intensity autocorrelation function *g* ^(2)^ for an ensemble of 90 µs pulse trains recorded on the SPB/SFX instrument at the European XFEL. Spatial intensity profiles were recorded at a shot-to-shot repetition rate of 1.128 MHz after photon transport through the SPB/SFX instrument.

Intensity distributions with time-dependent shot-to-shot statistics have previously been recorded during operation on the SPB/SFX instrument (Mancuso *et al.*, 2019 ▸) at the European XFEL. An example of the typical time-varying shot-to-shot variations in the wavefront intensity that are observed is shown in Fig. 1 ▸. These statistical fluctuations are in part due to processes specific to the operation of megahertz XFEL facilities, *e.g.* pulse distribution and parallel beamline operation, which are not well described by conventional models of XFEL radiation (Guest *et al.*, 2022 ▸). Time-dependent fluctuations in radiation intensity that occur within a pulse train can be described by the normalized two-time (auto-)correlation function *g* ^(2)^ (Goodman, 2000 ▸),

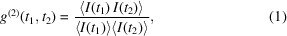

where the angle brackets denote the ensemble average over the 10 Hz pulse-train repetition rate. The pulse intensities *I* are mean centred with respect to the average intensity observed within their respective pulse train. This shot-to-shot correlation function is a measure of the stability in intensity between a pair of pulses observed at times *t*_1_ and *t*_2_, with *g* ^(2)^ = 1 denoting fluctuations in pulse intensity that are perfectly correlated, *g* ^(2)^ = 0 denoting no correlation and *g* ^(2)^ = −1 denoting anti-correlation. The shot-to-shot correlation of the pulse trains whose intensities are presented in Fig. 1 ▸ is provided in Fig. 2 ▸, demonstrating intensity statistics that are highly sensitive to the time of the recorded pulse.

These shot-to-shot correlations are emblematic of a dynamically evolving system (Madsen *et al.*, 2010 ▸) and illustrate that the shot-to-shot intensity statistics observed in an experiment are non-stationary. In this circumstance, the repeating checker-board pattern is representative of step-like changes in pulse intensity as a function of time (Bikondoa, 2017 ▸). In experiments where multiple diffraction patterns are integrated, the achievable spatial resolution (Gureyev *et al.*, 2008 ▸) is subsequently sensitive to the time at which the integrated pulses were recorded. These factors may play a significant role in determining the success of imaging experiments at the European XFEL, particularly in experiments making use of the high repetition rate to improve photon statistics or image microsecond dynamics.

## 3. A phenomenological model of XFEL wavefields

In this section, we present a method of replicating the photon statistics observed at the European XFEL. Using measurements of the pulse intensity recorded experimentally, we develop a model of the properties of the XFEL source wavefront. We map the statistics of fluctuations in the measured intensities to time-dependent stochastic variables of an analytical representation of the FEL source. In doing so, we are able to model arbitrary photon statistics without needing to take into account the underlying physical processes relating to their origins.

We construct a numerical representation of the source wavefield that is the product of two components: (i) a stationary Gaussian approximation of the fluctuations in the SASE pulse spectra (Saldin *et al.*, 2010 ▸) and (ii) a shot-to-shot fluctuating spatial envelope. The pulse wavefield at a distance *z* from the XFEL source, which we assume to be effectively fixed such that it has little impact on the properties of a pulse, is described by the complex scalar wavefield ψ(**r**, *t*), where **r** is the transverse position vector: **r** = (*x*, *y*). We write this wavefield as the superposition of two functions describing the transverse and longitudinal properties of the pulse wavefield, ψ_⊥_(**r**, *t*) and *f*(*t*). We assume the transverse properties to be a slowly varying function of time (microseconds) in comparison to the longitudinal components (femtoseconds), and subsequently treat each function independently. The pulse wavefield at a time *t* within a pulse train can be written as the Fourier integral

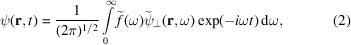

where 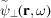 and 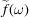 are the Fourier inverses of the transverse and longitudinal components of the pulse wavefield, respectively, and 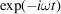 is the harmonic time factor.

Many coherent imaging applications are insensitive to changes in the pulse spectral distribution within the narrow spectral bandwidth of an FEL (Paganin & Pelliccia, 2021 ▸), and in many cases it is sufficient to approximate the linear SASE FEL process, such that the statistics of individual spectra are Gaussian (Saldin *et al.*, 2006 ▸). This enables us to make use of a computationally efficient representation of the pulse spectrum, which can be defined by Gaussian-filtered white noise (Pfeiffer, 2018 ▸). Following this approximate approach, here we define the SASE spectrum of each pulse using the procedure outlined in Appendix *A* [App appa].

### 3.1. Phenomenological description of pulse phase

Our model of the source wavefield is predicated on experimental observation. The pulse intensities observed at the European XFEL (Fig. 1 ▸) demonstrate time-dependent fluctuations in the measured wavefront intensity distribution. The source plane pulse wavefront is subsequently modelled using phase perturbations that are proportional to the fluctuations in the transverse centre of mass and beam width of intensities within a pulse train.

In modelling the transverse characteristics of the pulse wavefront, we make the assumption that shot-to-shot fluctuations in the source phase are the primary origin of fluctuations in the observed pulse intensities. This assumption implies both a constant pulse amplitude and a time-dependent beam phase. The evolution of the XFEL source intensity along the axis of propagation is determined by the gradient and curvature of the source wavefront phase (Teague, 1983 ▸). The phase gradient leads to shifts in the source intensity in the transverse plane, and the phase curvature leads to the convergence or divergence of the source intensity. We write the time-dependent transverse wavefield as a pulsed Gaussian beam with a time-dependent phase comprised of a linear prism term and a quadratic lens term (Saleh & Teich, 2019 ▸),

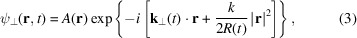

where *k* = 2π/λ_0_ for a central radiation wavelength λ_0_, and *R* and **k**_⊥_ = (*k*_*x*_, *k*_*y*_) are the radius of curvature and spatial wavevector in the transverse plane, respectively, which both vary with time. We define the time-constant amplitude factor with respect to the peak intensity of the pulse, *I*_0_, 

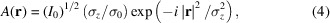

where σ_0_ and σ_*z*_ are, respectively, the beam widths at the waist and in a transverse plane located a distance *z* downstream of the waist.

### 3.2. Geometric approximation of fluctuations in the pulse phase

XFEL wavefields are highly paraxial. Estimates of statistics of the time-dependent components of the pulse wavefield *R* and **k**_⊥_ can therefore be obtained using geometric approximations of photon transport along the optical axis. The shot-to-shot fluctuations in the measured intensity profile at a distance Δ*z* downstream of a source emitting pulses of the form described in equation (3) are described by fluctuations in the tilt in curvature of the source.

Shot-to-shot phase tilts of the source plane wavefield lead to displacements in the time-dependent centre of mass (Δ*x*, Δ*y*) relative to the mean centre of mass calculated over all pulses within a pulse train. These displacements correspond to horizontal and vertical deflections of the pulse intensity,

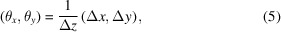

which are embedded in the pulse phase via its transverse wavevector,

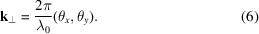

Similarly, fluctuations in the size of the beam by a factor Δσ can be used to infer fluctuations in the radius of curvature of the pulse wavefield around the train average 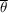 , 

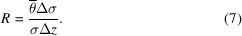

This geometric approximation of the spatial components of the source phase in our phenomenological model provides a route to extracting source-plane phase statistics from downstream measurements of the pulse intensity in the limit that pulses propagate along ray paths originating at the source.

## 4. Simulations and results

**Figure 3:**
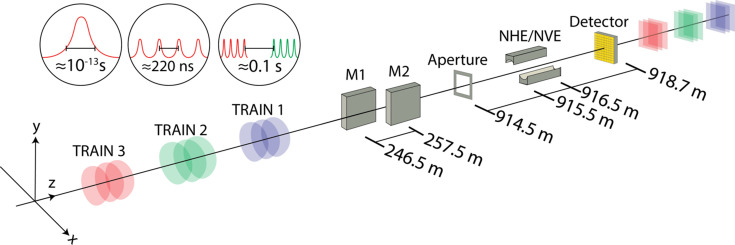
A schematic description of the experimental configuration of the SPB/SFX instrument at the European XFEL and (inset) the related pulse timescales. The beamline consists of two pairs of mirrors for photon transport and focusing: (i) a pair of horizontal offset mirrors (M1 and M2) and (ii) a pair of Kirkpatrick–Baez elliptical mirrors that focus the beam independently in the horizontal (NHE) and vertical (NVE) directions (Bean *et al.*, 2016 ▸). The focusing optics are preceded by a pair of beam-conditioning slits set to form a 3.8 mm square aperture in the transverse plane.

In order to replicate the shot-to-shot intensity correlations observed experimentally at the European XFEL (Fig. 2 ▸) we simulate stochastic pulse trains and their photon transport through the SPB/SFX instrument (Fig. 3 ▸).

Here we compare the output of wavefront propagation simulations using a phenomenological representation of the XFEL source with those obtained using wavefields from the FAST X-ray Pulse Database (Manetti *et al.*, 2019 ▸). Our goal is to demonstrate the effectiveness of our model by accurately reproducing the radiation statistics observed at the European XFEL, both on single-shot and on shot-to-shot timescales.

Pulse trains were simulated using a Python implementation of the phenomenological model outlined in Section 3. This implementation is PyPi installable and has been made available on Github: (https://github.com/twguest/phenom). The single-shot properties of each pulse were assumed to be Gaussian and of the form given in equation (3). Estimates of pulse width, divergence and energy were obtained using empirical models obtained from Sinn *et al.* (2011 ▸) as detailed in Appendix *B* [App appb].

### 4.1. Single-shot radiation properties

We simulated an individual XFEL pulse representative of the single-shot properties of the source by assuming no fluctuation in the beam pointing angle or curvature. The time-independent properties of the source were obtained from the empirical models provided in Appendix *B* [App appb] and were used to simulate pulses with a photon energy of 9.32 keV. These pulses were compared with pulses of the same energy obtained from the FAST Pulse Database after propagation through a numerical representation of the SPB/SFX instrument. The pulse width and divergence in the source plane were set to be 40.18 µm and 2.67 µrad, respectively, with a pulse energy of 0.52 mJ. We assumed a full width at half-maximum (FWHM) pulse duration of 25 fs and a spectral bandwidth Δω/ω = 1 × 10^−4^.

**Figure 4:**
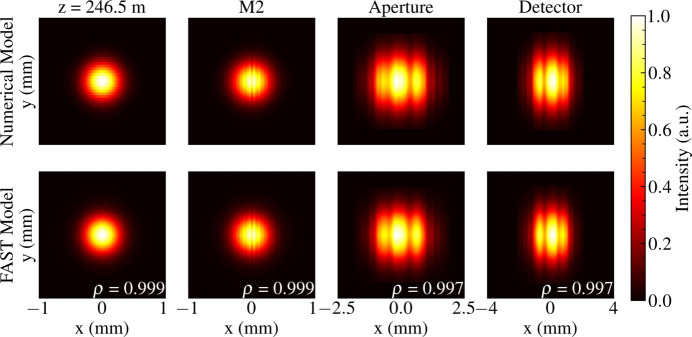
A comparison of simulated pulse intensities of the phenomenological model and pulses obtained from the FAST X-ray Pulse Database. The intensities are presented at multiple observation planes along the optical axis of the SPB/SFX instrument at the European XFEL. The Pearson correlation coefficient ρ between the intensity distributions calculated by each model is provided for each position along the beamline.

Propagation through the numerical representation of the SPB/SFX beamline (Yoon *et al.*, 2016 ▸) was implemented in *WavePropaGator* (*WPG*) (Samoylova *et al.*, 2016 ▸; Chubar *et al.*, 2013 ▸). A comparison of the simulated intensities of the time-independent pulse wavefield with the integrated pulse intensity of an ensemble of 100 FAST pulse wavefields is presented in Fig. 4 ▸.

The spatial intensity distributions of the mean pulse intensity predicted by our model accurately describe the mean pulse intensities calculated using the FAST XFEL source model. We observe an average Pearson correlation 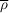 = 0.998 between intensity distributions obtained by propagating source wavefronts generated by our model and those generated by simulations based on fundamental FEL theory. The spatial structure of these intensity distributions is primarily determined by the surfaces and apertures of the photon transport optics (Pardini *et al.*, 2015 ▸). We expect that this time-independent Gaussian representation of the source wavefront provides accurate estimates of the characteristics of the irradiating wavefront in applications where the pulse intensity is observed after photon transport.

### 4.2. Shot-to-shot radiation statistics

The shot-to-shot fluctuations in the beam phase were simulated using the geometric approach described in Section 3.2. We simulated 100, 90 µs pulse trains at a photon energy of 6.0 keV, matching those in the experiment. The pulse duration and spectral bandwidth were again assumed to be 25 fs and Δω/ω = 1 × 10^−4^, respectively, while the pulse width, divergence and energy were set to 42.7 µm, 3.68 µrad and 0.79 mJ, respectively.

The phase of each pulse was determined using time-evolving probabilistic models describing the beam divergence and transverse pointing angles. Mean-centred fluctuations in the beam size and pointing angle were obtained from the recorded intensity distributions by calculating the beam width and centre of mass of each image individually. We determined the beam width to be the radius of the circle, with its origin at the beam centre of mass, enclosing 50% of the integrated pulse intensity. These measured quantities served as input data for our model, and the tilt and curvature of the phase of each pulse were defined under the geometric approximation described in Section 3.2 using probability distributions describing the magnitude of fluctuations in beam size and position as a function of time.

**Figure 5:**
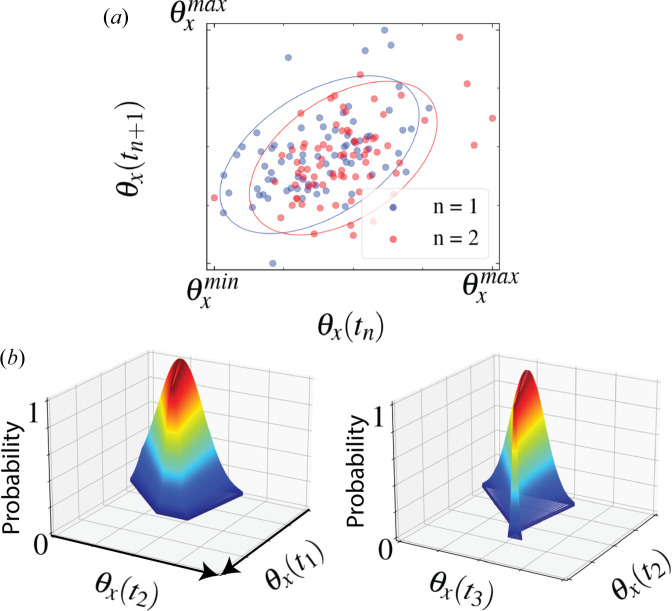
(*a*) A scatter plot of the statistical mapping process for the horizontal pointing angle θ_*x*_. By fitting an ellipse to the sample space of all of the recorded values for θ_*x*_ and times *t*_*n*_ and *t*_*n*+1_, we extract (*b*) a time-dependent probability distribution at time *t*_*n*+1_ that is dependent on the value at time *t*_*n*_. Truncation of the probability distribution along the θ_*x*_(*t* + 1) axis is due to the finite width of the covariance ellipse.

**Figure 6:**
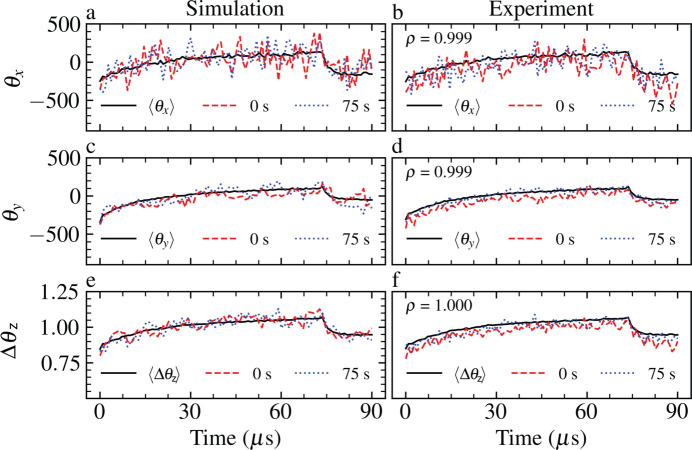
Pulse-train statistics (left-hand column) in the simulation and (right-hand column) from experiment. Mean values of the beam horizontal θ_*x*_, beam vertical θ_*y*_ and change in radius of curvature Δ*R* over a pulse train are given in black. Individual pulse trains are given for trains at 0 s (red dashed lines) and 75 s (blue dotted lines). The Pearson correlation coefficient ρ between the simulated and stochastic mean of each variable is given to three decimal places.

Probability distributions were obtained by fitting a covariance ellipse enclosing 95% of the recorded data in the state space of each pulse property at sequential time points *t* and *t* + 1 [Fig. 5 ▸(*a*)] (Schelp, 2018 ▸). These ellipses were used to define the mean and standard deviation of Gaussian probability distributions [Fig. 5 ▸(*b*)]. Using these probability distributions, the temporal properties of the beam phase were determined iteratively. Fig. 6 ▸ demonstrates the convergence of the stochastically generated parameters of the phenomenological model with experimental measurements.

**Figure 7:**
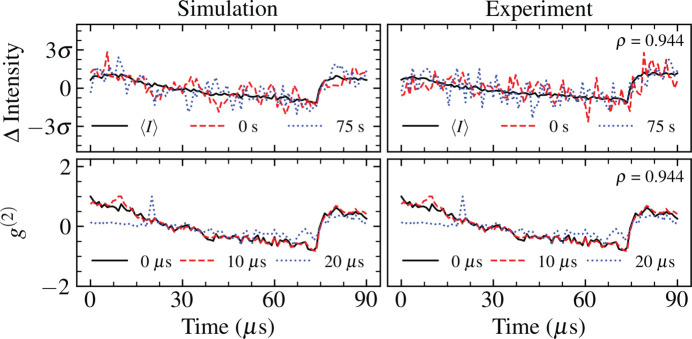
Pulse-train statistics (left-hand column) in the simulation and (right-hand column) from experiment. (Top) Mean values of the beam intensity over a pulse train are given in black. Individual pulse trains are given for trains at times 0 s (red dashed lines) and 75 s (blue dotted lines). (Bottom) Autocorrelation functions for pulses within a pulse train at times 0 µs (solid black lines), 10 µs (red dashed lines) and 20 µs (blue dotted lines). The Pearson correlation coefficient ρ between the simulated and stochastic mean of each variable is given to three decimal places.

Each pulse within a pulse train was propagated independently through the SPB/SFX beamline model to a detection plane 3.644 m downstream of the instrument focus, matching the parameters of the intensity measurements described in Section 2. A subset of the shot-to-shot intensities and intensity covariances of the simulated pulse trains after propagation through the SPB/SFX instrument are presented and compared with experimental measurements in Fig. 7 ▸.

**Figure 8:**
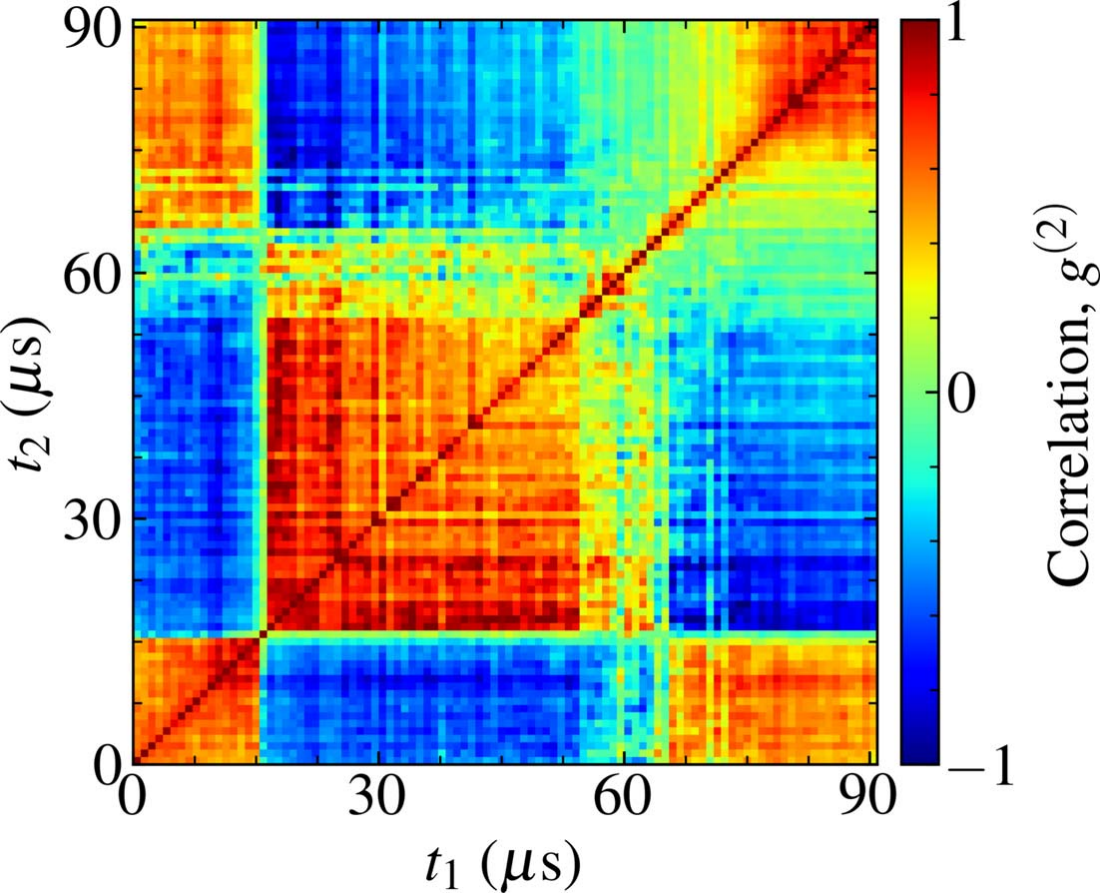
The two-time autocorrelation function *g* ^(2)^ of pulses generated using our phenomenological model.

We observe that the time-dependent characteristics of the experimental intensities are well described by the statistics of the simulated pulse trains after photon transport. Hence, by mapping the statistics of fluctuations in the width and centre of mass of experimental measurements of intensity to the analytical expression of the pulse phase in equation (3), we are able to produce numerical representations of the experimental pulse wavefront with fluctuations in intensity that are highly correlated with our observations. The autocorrelation function of the ensemble of pulse trains relative to the first pulse, *i.e.* *g* ^(2)^(*t*_1_ = 0, *t*_2_), is a linear function of the mean pulse intensity and thus shares the same highly correlated statistics. Such an autocorrelation function depicts the decay in the correlation between the first pulse in a pulse train and sequential pulses, which is of primary interest for time-resolved imaging experiments (Sun *et al.*, 2021 ▸). The full two-time autocorrelation function of the simulated pulse trains is presented in Fig. 8 ▸ and it is a reasonable description of the time-dependent intensity statistics observed experimentally (Fig. 2 ▸).

The intensity statistics of the experimental and simulated pulse trains are highly correlated, yielding an average Pearson correlation coefficient 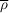 = 0.831. This high correlation suggests that the shot-to-shot fluctuations in pulse intensity observed experimentally can primarily be attributed to fluctuations in the phase of the XFEL pulse at the source. By expressing these phase fluctuations as geometric tilt and curvature terms, our phenomenological model enables accurate approximation of the experimental pulse train intensity statistics using intensity data as its input.

**Figure 9:**
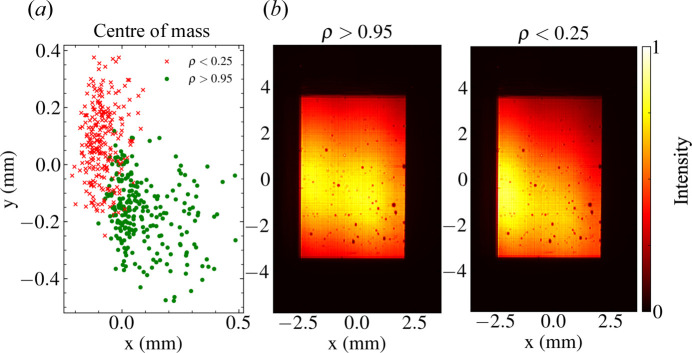
The centre of mass and integrated intensity of the input data as a function of the correlation between experimental and simulated pulse train statistics. (*a*) The distribution of the recorded centre-of-mass locations of pulse intensities observed experimentally. High correlation denotes recorded pulse intensities for which the geometric phase approximation of our model is suitable. Clustering of the centre of mass corresponding to simulated pulse trains with intensity statistics that are highly correlated (ρ > 0.95) and uncorrelated (ρ < 0.25) to those observed experimentally indicates the success and failure conditions of the model. (*b*) A comparison of the mean experimental intensity distribution used as input to the phenomenological model for these success and failure cases, illustrating that poor correlation arises from input data where significant truncation of the pulse intensity occurs.

The Pearson correlation between the experimental and simulated intensity statistics is time dependent and provides an indication of the conditions under which the geometric phase approximation of our model is suitable. Fig. 9 ▸ demonstrates that the magnitudes of the pulse pointing provided to the model for simulation are accurate when the beam makes a small deflection angle with the optical axis. We suggest that the primary limitation of this model is therefore the validity of the geometric phase approximation to the given input data.

Failure of the geometric phase approximation can occur when photon intensities are recorded downstream of the beamline optics. This occurs because a significant fraction of the pulse intensity falls outside the upstream mirror apertures. Fig. 9 ▸(*b*) illustrates that poorly correlated pulses are, on average, more truncated by the mirror aperture than highly correlated pulses. For a Gaussian beam truncated along one dimension, the true (prior to truncation) and recorded centres of mass deviate by a percentage beam width approximately equal to the percentage of intensity outside the mirror aperture. Consequently, the error in the calculated pointing angle increases linearly with pulse intensity losses (Appendix *C* [App appc]), notwithstanding redistribution of the incident intensity due to diffraction from the mirror edge. For the input experimental data used in our simulations, the maximum Pearson correlation ρ = 0.96 is reduced to ρ = 0.25 when approximately 16% of the radiant intensity lies outside the effective numerical aperture of the detector.

The overlap between the centres of mass of highly correlated and uncorrelated pulses in Fig. 9 ▸(*a*) provides an indication of beamline instabilities that are not currently captured by the SPB/SFX model. These instabilities, such as thermal deformation of mirror surfaces and positional jitter of photon transport optics, may result in a nonlinear relationship between the beam pointing angle and the observed centre of mass. While our results suggest that these phenomena are not the primary contribution to wavefront fluctuations on the megahertz timescale, these effects, which can arise over long-duration operations, may be significant contributors to the intensity statistics between pulse trains (Petrov *et al.*, 2022 ▸).

## 5. Discussion and outlook

Our phenomenological model of radiation at the European XFEL describes shot-to-shot fluctuations that are time dependent, and it requires no prior knowledge of the properties and operation of the accelerator and undulator. The mean intensity profile of our model is consistent with predictions based on FEL theory and, when paired with probabilistic models derived from experimental measurements, can be perturbed to replicate shot-to-shot wavefront statistics that these previous models do not describe. This model is a highly generalized approach to simulating XFEL radiation that can provide descriptions of the statistics of large ensembles of pulse wavefields under a broad range of operational conditions. The accuracy of these measurements is limited only by suitable estimates of the expected values of pulse width and divergence, and the quality of intensity data recorded. Our model provides a method of generating statistically accurate descriptions of the experimental pulse wavefield, in a manner that is robust to instrument operation and is suitable for users of XFEL facilities who may wish to carry out simulations of wavefront properties during an experiment.

When paired with efficient wavefront propagation simulations (Chubar & Celestre, 2019 ▸) our model provides a computationally efficient method for exploring the sensitivity of XFEL experiments to changes in the statistics of the shot-to-shot wavefield. Simulated pulses obtained from our model could play a critical role in estimates of the achievable resolution in serial XFEL experiments (Poudyal *et al.*, 2020 ▸; E *et al.*, 2022 ▸), which are inherently sensitive to the shot-to-shot temporal evolution of the pulse wavefield. Simulations of non-stationary pulse wavefields may provide new methods for interpreting common intensity artefacts that arise due to shot-to-shot fluctuations in the XFEL beam (Buakor *et al.*, 2022 ▸) and could improve the accuracy of simulations of key physical processes, such as X-ray-induced dynamics in samples and optics (Grünbein *et al.*, 2021 ▸; Kukk *et al.*, 2017 ▸; Zhang *et al.*, 2015 ▸; Abbey *et al.*, 2016 ▸). Under conditions in which the observed radiation statistics vary greatly from theoretical predictions, our model provides an opportunity to improve the technical design and commissioning of beamlines and optics (Williams *et al.*, 2017 ▸; Gaudin *et al.*, 2011 ▸).

Alongside the capacity to reproduce intensity distributions observed experimentally, our model provides new information on the shot-to-shot statistical properties of radiation at the European XFEL. In circumstances where the observed fluctuations in beam size and position are time dependent, our model predicts that the shot-to-shot coherence of the European XFEL is non-stationary. This implication of non-stationary spatial coherence between XFEL pulses has significant consequences in both imaging and wavefront characterization experiments, and is not captured in current descriptions of radiation at the facility (Geloni *et al.*, 2010 ▸). For large fluctuations in pulse properties, our model predicts that the quality of time-integrated diffraction data merged from large ensembles of pulses will be dependent on the index of the recorded pulses within their respective pulse trains. Consequently, improved reconstructions of the three-dimensional scattered intensity may be obtained by evaluating diffraction patterns obtained from coherent subsets of the source radiation, for example the subset of diffraction patterns produced by all *t*th pulses. In the case where pulse intensity or wavefront information can be recorded simultaneously during diffraction experiments, the development of filtering and classification algorithms (Rose *et al.*, 2018 ▸) that consider the non-stationary correlation between pulses may provide a route to improved outcomes in coherent diffractive imaging experiments.

Regarding the characterization and remediation of the pulse wavefront, non-stationary shot-to-shot coherence has significant implications in a broad range of applications. Measurements of coherence from intensity correlations, for example via the Siegert relation (Ferreira *et al.*, 2020 ▸), are not valid in this domain and single-shot methods such as grating interferometry should be applied instead (Makita *et al.*, 2020 ▸). Similarly, since fluctuations in the pulse wavefront can no longer be considered stationary ergodic, measurements of the XFEL wavefront obtained by integrating over multiple pulses, such as in ptychography (Daurer *et al.*, 2021 ▸), should be replaced in favour of single-shot methods (Sala *et al.*, 2019 ▸). We suggest that these predictions of the model be explored further using frameworks for separating stationary and non-stationary components of the shot-to-shot pulse wavefield (Manea, 2009 ▸), and they may benefit from the large volume of literature on pulsed correlation functions that have been developed to describe the coherence function of laboratory light sources (Dutta *et al.*, 2014 ▸, 2015 ▸).

Applications of our simplified representation of the XFEL source could prove highly beneficial as a photon diagnostic for the accelerator by providing a simple relationship between the stability of the observed intensities and the wavefront phase. This ‘top down’ approach may provide a route to optimizing operational parameters and could take advantage of machine-learning techniques in some cases to extract key factors of operation that determine FEL performance (Patel *et al.*, 2022 ▸). Following observations at the European XFEL and the Free-Electron Laser in Hamburg (FLASH) (Hellert & Schmidt, 2018 ▸), emphasis should be placed on the impact of electron-bunch orbit on the stability of the radiation properties. The origin of and sensitivity to non-stationary radiation statistics should be explored, and these factors are particularly relevant for future and developing high-repetition-rate facilities and upgrades (Raubenheimer, 2018 ▸; Hara *et al.*, 2021 ▸)

Finally, we expect that the residual disparity between the simulated and experimental shot-to-shot intensity fluctuations can be addressed in future applications by improving the experimental conditions under which fluctuations in the beam size and position are recorded. Specifically, it should be possible to achieve this by recording the shot-to-shot intensity distributions prior to photon transport. By further modifying the experimental setup used on the SPB/SFX beamline, our phenomenological model can be readily extended to represent shot-to-shot statistics of other pulse properties, including pulse energy, duration and spectra. This could be achieved using currently available photon diagnostics (Grünert *et al.*, 2022 ▸; Kujala *et al.*, 2020 ▸) and may provide the opportunity to describe pulse characteristics in more complex modes of operation. This includes circumstances where the transverse and longitudinal components of the pulse wavefield cannot be considered independent.

We note that our model is intended to complement solutions based on theoretical descriptions of the SASE radiation process. For applications that are highly sensitive to pulse spectra, this model should be used in conjunction with these methods to describe highly nonlinear modes of operation.

## 6. Conclusions

We have presented a phenomenological model of shot-to-shot wavefront statistics at the European XFEL. Our model maps the shot-to-shot statistics of intensity fluctuations observed experimentally to a geometric representation of the source phase. Unlike alternative models of XFEL radiation, our approach is robust with respect to non-fundamental practical effects caused by instrument operation, while requiring no prior knowledge of their physical origins. Using wavefront propagation simulations, we have demonstrated the capacity of our model to represent pulse wavefronts that reflect the time-dependent intensity statistics observed at the European XFEL.

## Figures and Tables

**Figure 10:**
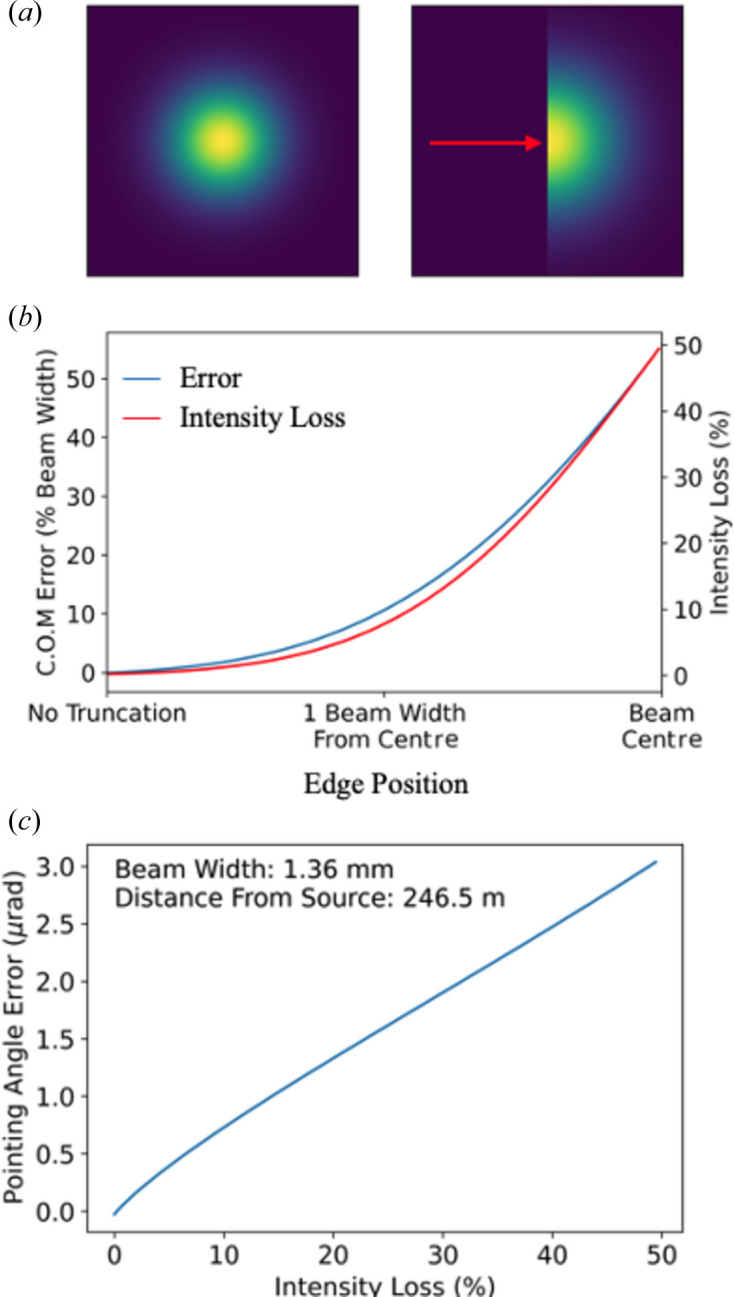
Evaluation of the error in the recorded beam pointing angle due to truncation by an aperture. (*a*) We simulate a Gaussian pulse intensity that is tilted towards a one-dimensional binary mirror edge, denoted by the red arrow. (*b*) With increasing pointing angle, the mirror edge intersects different points of the beam intensity; all pulse energy outside this mirror edge is lost. (*c*) Simulations of a 9.32 keV beam described by the parameters of our empirical model, intersecting the HOM1 mirror of the SPB/SFX instrument.

## Appendix A SASE spectrum model

We obtain the complex SASE spectrum  by first defining an electric field spectrum  ,

where *g*(ω) is the complex spectral envelope of the XFEL beam modulated by a stochastic phase term ϕ_0_(ω) of frequency ω. We define the spectral envelope to be Gaussian and real-valued,

The characteristic stochastic SASE FEL temporal pulse profile is a complex scalar wavefield which we approximate as the product of the scalar Fourier inverse *f*_0_(*t*) of equation (8) and a real-valued temporal envelope *h*(*t*),

where the temporal envelope of the pulse is centred at time τ, with a width equivalent to the FWHM duration of the radiation pulse, *t*_0_ = 25 fs,

Using the inverse Fourier transform, we may write *f*(*t*) as

which forms a Fourier pair with the fast-timescale complex spectral envelope in equation (2),

## Appendix B Empirical model parameters

Estimates of pulse divergence, width and energy were obtained using empirical models formulated in terms of photon energy *E*_0_ (in electronvolts) and electron beam charge (Sinn *et al.*, 2011 ▸). These expressions are:

The pulse energy *U* is included in the model via the peak radiation intensity *I*_0_ in equation (4). For a Gaussian beam, these properties are related thus:

where *t*_0_ is the pulse duration.

## Appendix C Centre of mass of a Gaussian beam at an aperture

Our phenomenological model makes the assumption that fluctuations in the centre of mass of recorded intensity distributions between pulses are due to plane-wave pointing angle tilts at the source. This method enables the beam phase to be extracted from intensity measurements, under the requirement that the pulse centre of mass follows a ray path. This is largely true for paraxial optics, but the approach is limited in its description of beams that are significantly obstructed or deformed by optics prior to measurement.

Fig. 10 ▸ indicates that the percentage loss in beam intensity at a one-dimensional aperture is approximately equal to the error in the calculated beam centre of mass as a percentage of beam width. Applying this to the study of pulse parameters relevant to the SPB/SFX instrument of the European XFEL, we note that the beam pointing angle error scales linearly with the intensity loss due to truncation of the beam.
